# Non-targeted metabolomics-based molecular networking enables the chemical characterization of *Rumex sanguineus,* a wild edible plant

**DOI:** 10.1007/s11306-024-02210-2

**Published:** 2025-01-24

**Authors:** Valentina Ramundi, Mitja M. Zdouc, Enrica Donati, Justin J. J. van der Hooft, Sara Cimini, Laura Righetti

**Affiliations:** 1 Laboratory of Organic Chemistry, Wageningen University & Research, 6708 WE Wageningen, the Netherlands; 2 Institute for Biological Systems (ISB), National Research Council of Italy (CNR), Via Salaria Km 29.300, Monterotondo Scalo, 00015 Rome, Italy; 3 Department of Science and Technology for Humans and the Environment, Università Campus Bio-Medico di Roma, Via Álvaro del Portillo, 21, 00128 Rome, Italy; 4 Bioinformatics Group, Wageningen University & Research, Droevendaalsesteeg 1, 6708 PB Wageningen, the Netherlands; 5 Department of Biochemistry, University of Johannesburg, Johannesburg, 2006 South Africa; 6 National Biodiversity Future Center, NBFC, 90133 Palermo, Italy; 7 Wageningen Food Safety Research, Wageningen University & Research, 6700 AE Wageningen, the Netherlands; 8 Present Address: Helmholtz-Munich Metabolomics and Proteomics Core Facility (MPC) , Ingolstädter Landstraße 1, Buildings 25 and 34, 85764 Munich, Neuherberg Germany

**Keywords:** Wild edible plants, Specialized metabolites, Polyphenols, Emodin, Feature-based molecular networking, Metabolomics

## Abstract

**Introduction and Objective:**

*Rumex sanguineus*, a traditional medicinal plant of the *Polygonaceae* family, is gaining popularity as an edible resource. However, despite its historical and nutritional significance, its chemical composition remains poorly understood. To deepen the understanding of the of *Rumex sanguineus* composition, an in-depth analysis using non-targeted, mass spectrometry-based metabolomics was performed.

**Methods:**

Rumex roots, stems and leaves samples were analyzed by UHPLC-HRMS and subsequently subjected to feature-based molecular networking.

**Results and Conclusion:**

Overall, 347 primary and specialized metabolites grouped into 8 biochemical classes were annotated. Most of these metabolites (60%) belong to the polyphenols and anthraquinones classes. To investigate potential’ toxicity due to the presence of anthraquinones, the amount of emodin was quantified with analytical standard, revealing higher accumulation in leaves compared to stems and roots. This highlights the need for thorough metabolomic studies to understand both beneficial and harmful compounds, especially in plants with historical medicinal use transitioning to modern culinary use.

**Supplementary Information:**

The online version contains supplementary material available at 10.1007/s11306-024-02210-2.

## Introduction

By 2050, the world population is predicted to have grown to 10 billion people, and, as a consequence, the food demand will dramatically increase. Over the past few decades, agriculture has primarily focused on enhancing crop productivity to meet the growing demand for food. However, the innovative farming techniques may not be sufficient to guarantee market requirements in the future. Moreover, today more than ever, great attention is paid not only to agricultural yield but also to food quality. We know more than 50,000 plants that are considered edible, but only few of them have been cultivated as food crops (The Plants That Feed the World, 2023). In this scenario, the utilization of wild edible plants (WEPs) can have an interesting role in making the food system healthier, sustainable and resilient to the current climate changes. WEPs gathering has been a habitual practice since ancient times all over the world, however they continue to attract the scientific interest for different reasons even now. Indeed, WEPs are a good source of specialized/secondary metabolites, which can have health-promoting effects (Ceccanti et al., 2018) and are generally more tolerant to adverse climate conditions in comparison to crops. The identification of effective sources of stress tolerance is an important aspect for an effective plant protection strategy. In this sense, wild plants constitute a rich natural reservoir of genes involved in plant stress tolerance. Despite this, WEPs remain understudied, leading to a scarcity of knowledge regarding their bioactive constituents and nutritional properties. In particular, the genus of *Rumex*, belonging to the polygonaceae family, includes approximately 200 species and it is notable among the herbaceous plants of temperate climatic zones. In fact, *Rumex* species have several advantages, such as a short vegetative period, rapid biomass accumulation, and adaptable growth requirements (Van Assche et al., 2002). Plants belonging to *Rumex* genus are also great producer of phenolic compounds thus making these plants interesting source of bioactive molecules (Mishra et al., 2018). Moreover, several plants belonging to *Rumex* genus are also included in the pharmacopoeias of different countries (Ernst, 2012; Shaikh et al., 2018; Shikov et al., 2021). In Europe, *Rumex acetosa* and *Rumex acetosella* (Korpelainen & Pietiläinen, 2020) are routinely incorporated into dietary practices whereas *R. sanguineus,* commonly known as bloody dock or red-veined sorrel, still remains relatively underexplored in terms of nutritional values and health benefits (Feduraev et al., 2022). Leaves were traditionally consumed fresh or cooked. While the tender, young leaves are enjoyed in their raw state as a fresh, leafy vegetable, the mature leaves require blanching, due to the presence of oxalic acid in adult leaves, which may cause adverse effects.

**Fig. 1:**
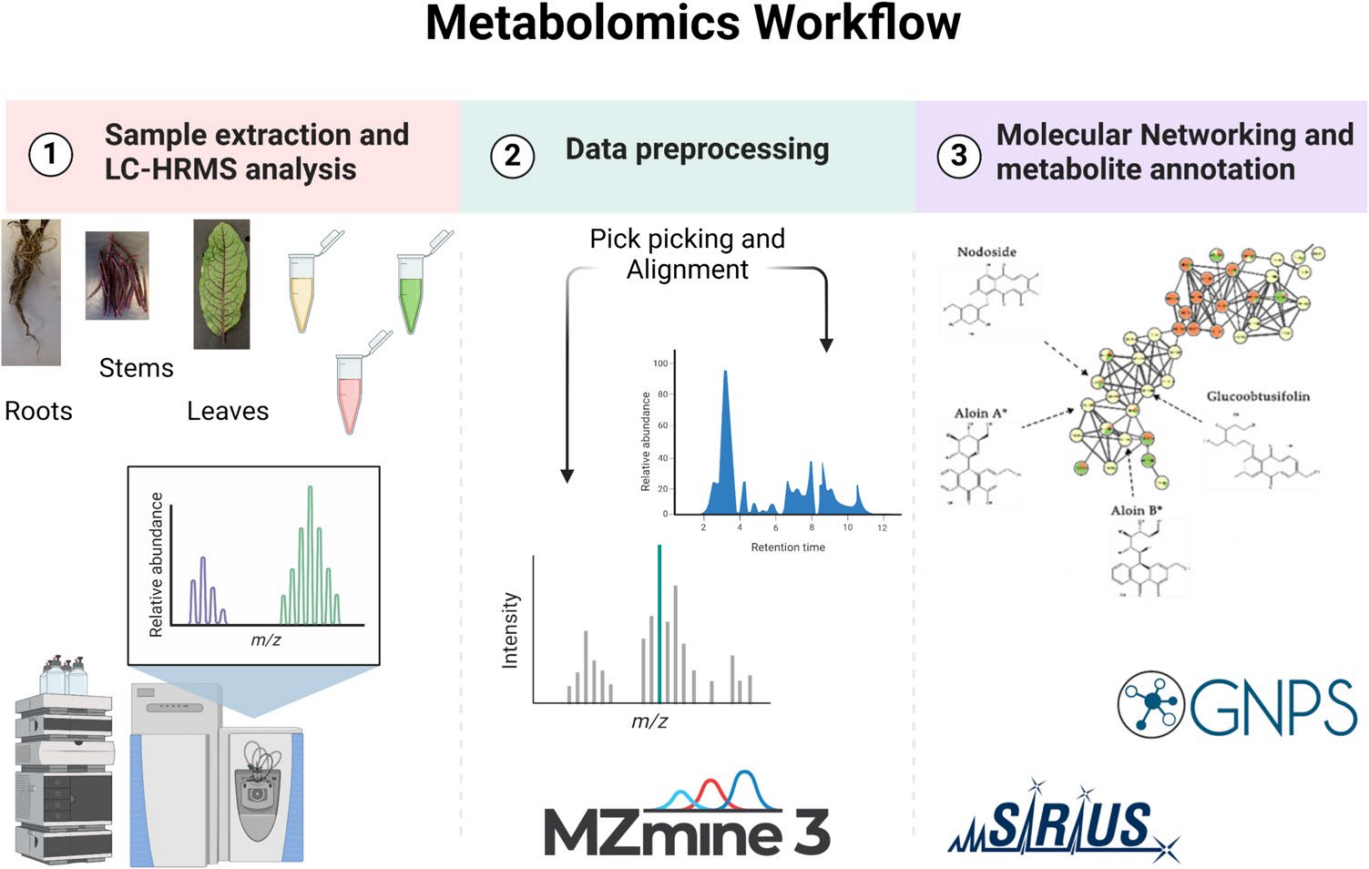
Schematic representation of the workflow of the study: (1) extraction and instrument analysis of roots, stems and leaves of *R. sanguineus* by LC-ESI-HRMS; (2) data pre-processing with MZmine: pick picking and features alignment; (3) building of a feature-based molecular network on GNPS (Global Natural Product Social Molecular Networking) and structural elucidation of the annotated metabolites with SIRIUS. Created in BioRender. Ramundi, V. (2024) https://BioRender.com/l93d646

While there is scientific evidence to support the traditional use (both medicinal and culinary) of many *Rumex* species (Jeon et al., 2022) less is known about its metabolic profile. Scientific evidences are available regarding its content of anthocyanins vitamin C, iron, and other minerals (Feduraev et al., 2022; Vasas et al., 2015) However, a comprehensive characterization of the secondary metabolites (Panche et al., 2016) of the different organs of this plant, roots, stems and leaves, is yet to be uncovered (Korpelainen & Pietiläinen, 2020). Up to now, targeted methods, developed for quantification of a given class of metabolites, have been exclusively applied to investigate *R. sanguineus* chemical composition (Feduraev et al., 2022), mainly focusing on a restricted metabolite’s class. Nevertheless, nowadays, advanced analytical tools permit the simultaneous analysis of hundreds of metabolites, allowing a better characterization of small molecules (up to 1200 Da), therefore, the composition of complex plant matrices can be investigated in depth. In fact, in the last decade, the applicability of metabolomics to food science and nutrition research has strongly emerged (Charria-Girón et al., 2023; Zdouc et al., 2021). However, one of the major bottlenecks of the non-targeted metabolomics workflow is the annotation step. Indeed, on average, only 5% of the aligned features can be annotated, while the 95% remains unknowns, limiting our view of the metabolome. Recently, several approaches to improve feature annotation have been proposed (Aron et al., 2020; Zdouc et al., 2021). Among these tools, Feature-Based Molecular Networking (FBMN) (Watrous et al., 2012) has emerged as computational approach to improve feature annotation and visualization of the relationships between different compounds based on their structural similarities. The basic idea behind classical molecular networking (MN) is to group together features with MS/MS spectra that have similar mass fragmentation patterns, and with each feature represented as a node in a network, construct clusters or families of chemically-related features (Nothias et al., 2020). FBMN improves upon classical MN by leveraging MS1 information such as isotope patterns and retention time. This enables the differentiation of isomers with similar MS2 spectra and facilitates the inclusion of relative quantitative data for robust downstream metabolomics statistical analysis. Additionally, FBMN aids in data redundancy reduction that simplifies the discovery of structurally related compounds. The added value of this approach has been already demonstrated in different metabolomics application fields (e.g. natural products, food safety, (Li et al., 2020)) leading to an increase in annotated compounds up to 10% (de Jonge et al., 2022; Wang et al., 2016). In this study, we aimed to take advantage of the potential of such a powerful approach, schematically represented in Fig. 1, to comprehensively characterize the chemical profile of *R. sanguineus*.

## Materials and methods

### Chemicals and reagents

LC–MS grade methanol and acetonitrile were purchased from Biosolve Chimie (Netherlands). Bi-distilled water was obtained using Milli-Q System (Millipore, Bedford, MA, USA), formic acid (CAS 64-18-6), from Fisher Chemical (Thermo Fisher Scientific Inc., San Jose, CA, USA) was also used. The following standards were purchased from Sigma-Aldrich Chemie GmbH (Germany): emodin (CAS 518-82-1) emodin-8-glucoside (CAS 23313-21-5), aloin A (CAS 1415-73-2), aloin B (CAS 28371-16-6) and L-triptofan-d5 (CAS 62595-11-3). Fluka Chemie GmbH (Buchs, Switzerland) provided rutin (CAS 153-18-4). Kaempferol (CAS 520-18-3) and quercetin (CAS 849061-97-8) were obtained from PhytoLab GmbH & Co. KG (Germany).

### Plant material

For this study*, Rumex sanguineus* seeds were stratified at 4 °C in dark for 3 days before sowing them on soil. Seeds (2–4 per pot) were sowed in pots (8 × 8 cm in size) in a 6:1 mix of soil and perlite, germinated and grown at + 25 °C in the C.N.R. (National Research Council) greenhouse in Montelibretti (Monterotondo, Italy), following the protocol previously reported (Van Assche et al., 2002). Subsequently, seedlings were transferred to larger pots (30 × 40 × 20 cm), where 28 plants grew for 1 year until the end of the experiment. The pots were kept at 40% humidity, 16/8 h photoperiod at + 25 °C in the greenhouse for thirteen months. The watering of the plants was repeated every 3 days. During the vegetative stage, one year after germination, the initial sampling was conducted. At this stage, adult leaves were collected separately from young leaves, with adult leaves measuring between 7.34 and 15.27 cm, and young leaves measuring between 2.47 and 7.34 cm (Figure S1). In this first stage, 3 biological replicates each for old and young leaves were collected. For the subsequent sampling, adult leaves, stems and roots were collected to obtain 3 biological replicates for each organ. After each sampling, the plant material was frozen using liquid nitrogen, grounded into a homogeneous and fine powder and freeze-dried for 72 h.

### Non-targeted metabolomics: sample extraction and LC-HRMS analysis

Sample extraction of roots, stems and leaves was performed as previously reported (Salem et al., 2016). Briefly, 25 mg of homogenized tissues were extracted with 1650 µl of extraction solvent mix (water, methanol, methyl tert-butyl ether). After centrifugation, 600 µl of supernatant were dried down under nitrogen flow and then reconstructed in 300 µL UPLC-grade methanol/water (1:1, vol/vol). 100 µL of the extract was transferred into a vial and added with the internal standard at 1 mg/L concentration (L-tryptophan d-5). To measure performance and system stability and assess the reproducibility of the sample treatment procedure, Quality Control samples (QC) were injected during the analyses. QCs were obtained by mixing equal volumes (10 µL) of all 24 sample extracts and following the same procedure as for the other samples. QCs were injected at the beginning of the run and after every 8 real samples.

The data acquisition was performed using Agilent 1290 UHPLC system coupled to a Thermo Fisher Q-Exactive Focus Quadrupole-Orbitrap Mass Spectrometer equipped with a heated electrospray ionization (HESI) interface. 24 samples were analyzed, comprising 4 biological replicates extracted in duplicate (8 roots, 8 stems and 8 leaves). Sample extracts were injected (5 µL) and chromatographically separated using a reversed-phase C18 HSS T3 Acquity column 2.1 × 100 mm, 1.8 µm particle size (Waters, Milford, MA, USA). The mobile phases used for chromatographic separation were water containing 0.1% formic acid (Buffer A) and acetonitrile containing 0.1% formic acid (Buffer B). A gradient profile was applied using water (eluent A) and acetonitrile (eluent B), both acidified with 0.1% formic acid as mobile phases. A multi-step elution dual-mode gradient was developed as follow: at 0.0 min (5% B; 0.40 mL/min) a gradient begun as follows: 11 min 35% B, 12.5 min 70% B, 13.5 min 99% B, held for 1.5 min until 15.0 min, and then in 1 min the initial conditions were restored to 5% B; the total run time was 21 min including 5 min of re-equilibration. The column was maintained at 40 ℃ and a flow rate of 0.4 mL/min used. The settings of the interface, HESI source, were configured to operate effectively in both negative and positive ion modes, and the parameters were set as follows: sheath gas flow rate 30 mL/min, aux gas flow rate at 15 mL/min and sweep gas flow rate at 33 mL/min, spray voltage 3.2 kV, capillary temperature 320 ℃, S-lens RF level 47.0, aux gas heater temp 0 ℃. The mass spectra were acquired in DDA (Stincone et al., 2023) (Data Dependent Analysis) by full scan MS1 and MS2 in positive and negative ionization mode on a Q-Exactive high resolution Orbitrap-type MS (ThermoFisher, Bremen, Germany) in a scan range from 100 to 1000 m*/z*; the full MS resolution was 70,000 FWHM, AGC target 3e6, maximum IT 200 ms and with a profile spectrum data type. The dd/MS^2^ analysis was performed with a resolution of 17,500 FWHM with three different collision energy: 15,30,45 eV, AGC target 2e5, maximum IT 50 ms, loop count of 3, minimum AGC target of 8.00e3, intensity threshold 1.6e5, apex trigger 2 to 15 s, dynamic exclusion of 4.0 s, charge exclusion 2–8, > 8 and a profile spectrum data file.

### Non-targeted metabolomics: multivariate data analysis and metabolite annotation

Multivariate data analysis was conducted using Metaboanalyst 5 (Pang et al., 2021), with the aim of confirming the absence of any anomalies in the instrumental analysis process, thus enhancing the overall accuracy and reliability of the data collected. The data set was normalized on the internal standard (L-tryptophan d-5), and then principal component analysis (PCA) score scatter plots were obtained, as depicted in Fig. S2. Data acquired in the RAW Thermo format, were converted to.mzXML using Proteowizard’s MSConvertGUI (64-bit) tool (Chambers et al., 2012). Converted.mzXML data were then imported to MZmine 3.9.0 (Katajamaa et al., 2006), to perform data pre-processing using ADAP (Automated Data Analysis Pipeline) including EIC (Extracted Ion Chromatogram) construction, chromatographic peak detection, spectral deconvolution and alignment with the following parameters: (1) Mass detection = retention time, auto, MS1 noise level 5.0E3, MS2 noise level 1.0E3. (2) ADAP chromatogram builder = retention time 2.00–15.00 min, MS1 level minimum consecutive scans 8, minimum intensity for consecutive scans 2.0E4, minimum absolute height 3.0E4, *m/z* tolerance scan-to-scan 5.0 ppm; (3) Smoothing = Savitzky Golay; (4) Local minimum feature resolver = minimum relative feature height 25%, chromatographic threshold 75%, Minimum search range RT/Mobility 0.050, minimum absolute height 5E4, peak duration 0.10–3.00 min; (5) Isotopic filtering = retention time tolerance 0.020 s, *m/z* tolerance 0.0010 or 5.0 ppm, monotonic shape no, maximum charge 2, representative isotope, most intense; (6) Isotopic peaks finder = m/z tolerance 0.0010 or 5.0 ppm, maximum charge of isotope *m/z* 1; Join aligner = 0.0015 or 5.0 ppm, weight for m/z 3, retention time tolerance 0,200 min, weight for RT 1, (7) Feature list rows filter = Minimum aligned features 1, retention time filter 2.00–15.00 min, Chromatographic FWHM (Full Width at Half Maximum) 0.00–6.00, never remove feature with MS2, on; (8) Peak finder = intensity tolerance 25%, *m/z* tolerance 0.002 or 5.0 ppm, retention time tolerance 0.200 min, minimum scans 4. Features with retention time below 2.0 min and above 15.00 min were excluded.

The resultant feature list comprised 2060 features for ESI positive ion mode and 1118 for ESI negative ion mode, were exported to a format compatible with GNPS and SIRIUS (.csv and.mgf with a metadata file) utilizing the dedicated "Export for GNPS" or “Export for SIRIUS” feature provided within the software.

A Feature-Based Molecular Networking (Nothias et al., 2020) (FBMN) version, (Release_27), was generated by exporting the output data from MZmine 3.9.0. and uploading.mgf,.csv, and metadata files onto the GNPS platform. The parameters employed for generating the Feature-Based Molecular Network are as follows: precursor ion mass tolerance of 0.01 Da, fragment ion mass tolerance 0.01 Da, minimum cosine score that must occur between a pair of MS/MS spectra in order to form an edge in the molecular network of 0.70, minimum number of fragment ions that are shared between pairs of related MS/MS spectra, in order to be connected by an edge in the molecular network of 7, maximum shift between precursors 500 Da, maximum number of neighbour nodes for one single node of 20, maximum size of nodes allowed in a single connected network 100; search analogs, on, minimum number of fragments that MS/MS spectra should contain in order to be considered for annotation of 6, score threshold of 0.7, maximum analog search mass difference 100 Da, filtering options, off. The created FBMN was then downloaded as a Cytoscape (Shannon et al., 2003) file and then visualized using the same tool (Cytoscape 3.10.1). The FBMN job of the positive ionization mode dataset is publicly available at https://gnps.ucsd.edu/ProteoSAFe/status.jsp?task=f3363189286c428bae3470d9fbd371c6, while the FBMN job of the negative ionization mode dataset is available at https://gnps.ucsd.edu/ProteoSAFe/status.jsp?task=4e3d798eb99e42a58f0401ca78399186. The MS data were deposited on public repository (Dataset: MSV000092024), https://massive.ucsd.edu/ProteoSAFe/dataset.jsp?accession=MSV000092024. This approach enabled us to visually organize spectral data and gain insights into the distribution of the same compound among the three distinct organs.

All the detected features were computed using SIRIUS 4 both in positive and negative ionization modes with the following parameters: mass accuracy 5 ppm (Orbitrap), all possible adducts; ZODIAC (Identification of molecular formulas) use 2 step approach, edge thresholds 0.95, min local connections 10; CSI:FingerID (predicts molecular fingerprint from MS/MS and fragmentation tree for each compound) all fallbacks adducts, search molecular structure in all databases, tag lipids, off; CANOPUS (predicts compounds categories for each compound individually predicted based on its predicted molecular fingerprint) on.

The identification level of the annotated metabolites is reported following the Chemical Analysis Working Group (CAWG)(Sumner et al., 2007) criteria, including level I (comparison against an authentic standard) and level II (putatively identified molecule) annotations (for a full list, see Zenodo upload https://doi.org/10.5281/zenodo.14236385). For the annotation, both spectral matching (comparison with identified spectra, in Biocyc, Pubchem, HMDB, COCONUT, CHEBI, KEGG, Natural Products, SuperNatural) and literature search were used.

### Targeted analysis of anthraquinones

With the aim to quantify the amount of emodin and emodin 8-glucoside, samples were extracted following the protocol previously described by Shi et al., 2018 (Shi et al., 2018). Briefly, 10 mg of roots, stems and leaves were extracted with 1 ml of LC-grade methanol/water (1:1, vol/vol) followed by ultrasonication for 30 min. Samples were then centrifuged (14,000 rpm, 5 min, 4 °C) and the supernatants were collected and diluted (100-fold dilution) prior to UHPLC-HRMS analysis. Calibration curves were set up using external standards (range 0.05 mg/L to 1 mg/L for emodin and 0.01 mg/L to 0.5 mg/L for emodin-8-glucoside) for target analyte quantification. Satisfactory linearity was obtained for each compound (emodin R^2^ = 0.9996, y = 6E + 06x + 222,814; emodin-8-glucoside R^2^ = 0.9993, y = 1E + 07x − 17,982). Data acquisition was performed by Thermo Xcalibur 2.2 software (Thermo Fisher Scientific, Waltham, MA, USA). A one-way ANOVA with a p-value cutoff of 0.05 followed by a Tukey’s HSD post hoc was performed using XLStat to determine statistically significant accumulation of emodin and its glucoside across root, stem and leaf samples.

## Results and discussion

Extracts from the roots, stems and leaves, of *R. sanguineus* were subjected to non-targeted metabolomics workflow to comprehensively annotate its metabolites composition. The water/methanol phase allowed for the extraction of the main plant biochemical classes of both primary (i.e. carbohydrates) and specialized metabolites (i.e. flavonoids, anthraquinones). The resulting UHPLC-HRMS raw datasets, acquired both in positive and in the negative ionization modes, underwent several data processing steps, as schematically represented in Fig. 1. Overall, 8896 and 10,589 features were aligned in positive and negative mode respectively, using MZmine 3.9.0. At first, unsupervised models were employed, specifically PCA, to reduce the dimensionality of our dataset while maximizing the retention of its inherent variability. The PCA score plots (depicted in Figure S2 of supplementary information) reveals different metabolomic composition for *Rumex* organs, due to a distinctive clustering pattern among the three different organs. Afterwards, the aligned datasets (ESI positive and negative) were independently analyzed using the feature-based molecular networking workflow of GNPS and visualized using Cytoscape software. The resulting molecular networks are reported in Figures S3, S4 for ESI positive and negative ion modes, respectively.

### Overview of metabolite annotations: identification levels, biochemical groups, and tissue distributions

Overall, the annotation process in both positive and negative ionization mode yielded 449 annotated features, including 102 overlapping annotations, resulting in 347 unique metabolites. Of these, 200 metabolites were annotated in positive ESI mode, and 143 metabolites were annotated in negative ESI mode. Within all the annotated features, 2% (7) were identified at level I (Schymanski et al., 2014) indicating the highest confidence level of identification, while 98% (442) were categorized as level II, denoting a reliable matching with existing libraries. The lists of annotated features are uploaded to Zenodo: https://doi.org/10.5281/zenodo.14236385.

**Fig. 2:**
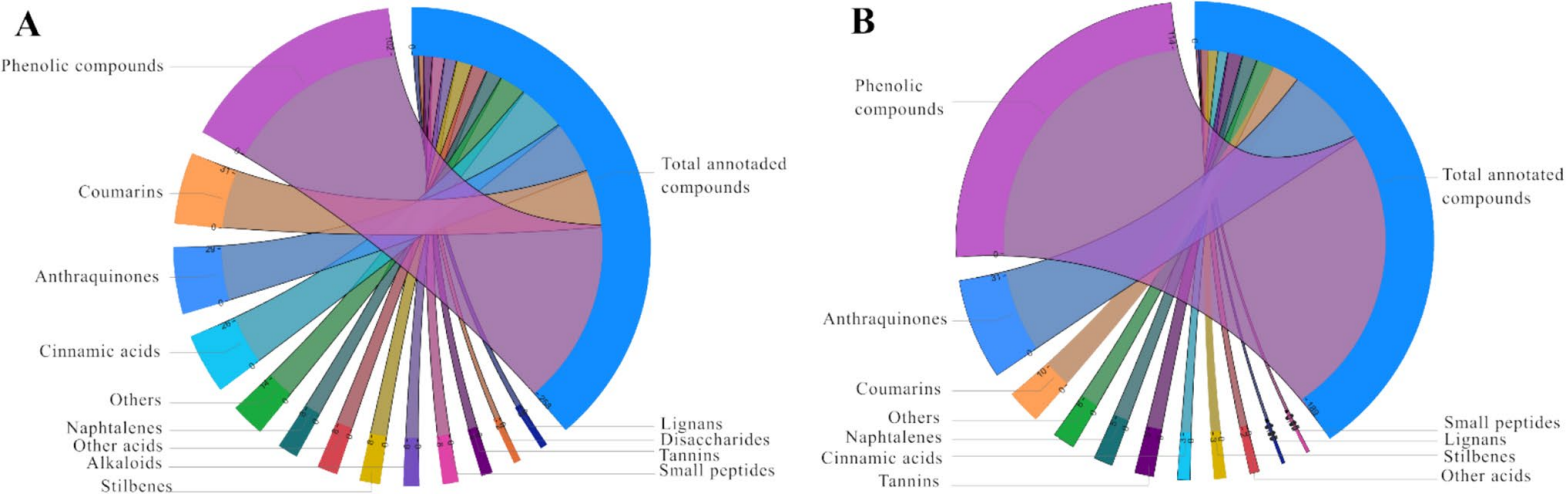
Distribution in different chemical classes of the 347 annotated compounds, of which 200 annotated in the positive ESI ionization mode (**A**) and 143 in the ESI negative ionization mode (**B**). In both instances, the predominant category among the annotated compounds comprises phenolic compounds

The 347 primary and specialized metabolites were grouped into 8 biochemical classes, as depicted in Fig. 2. This categorization offers a comprehensive snapshot of the chemical diversity within the analysed compounds under different ionization modes. As an example, some chemical classes such as alkaloids and disaccharides ionize exclusively in positive mode while polyphenols ionized mainly in negative mode. Among the annotated compounds, well-established constituents found in other *Rumex* species, such as polyphenols and anthraquinones, were found. In addition, our analysis unveiled a significant presence of coumarins, surpassing what is typically reported in literature for other *Rumex spp.* (Vasas et al., 2015). Notably, coumarin presence has been rarely reported in *Rumex* species, with only *R. roseus* (Saoudi et al., 2021) showing some derivatives. Several cinnamic acids were also annotated. These antioxidant compounds were previously identified and found responsible for the biological activity of the *R. dentatus* extract (Khaliq et al., 2023).

However, most annotated metabolites (60%) still belong to the polyphenols and anthraquinones classes. Cyanidins and proanthocyanidins were previously found only in *R. acetosa* (Bicker et al., 2009). Flavonoids, which are prevalent in the leaves of the *Rumex* genus, are primarily renowned for their beneficial attributes (Rana et al., 2022). A wide range of glycosylated quercetin derivatives like rutin were annotated in this study. Furthermore, while flavonol derivatives like kaempferol are present in subgenus species such as *R. confertus* and *R. sanguineus*, they are notably absent in *R. acetosa* and *R. acetosella*. Kaempferols and quercetins play a crucial role as health promoters due to their potent antioxidant properties and their potential to mitigate oxidative damage in biological systems (Zhang & Tsao, 2016). Therefore, the accumulation of these bioactive phytochemicals in the edible part of *R. sanguineus* underscores its significance as a food source of these beneficial compounds.

**Fig. 3:**
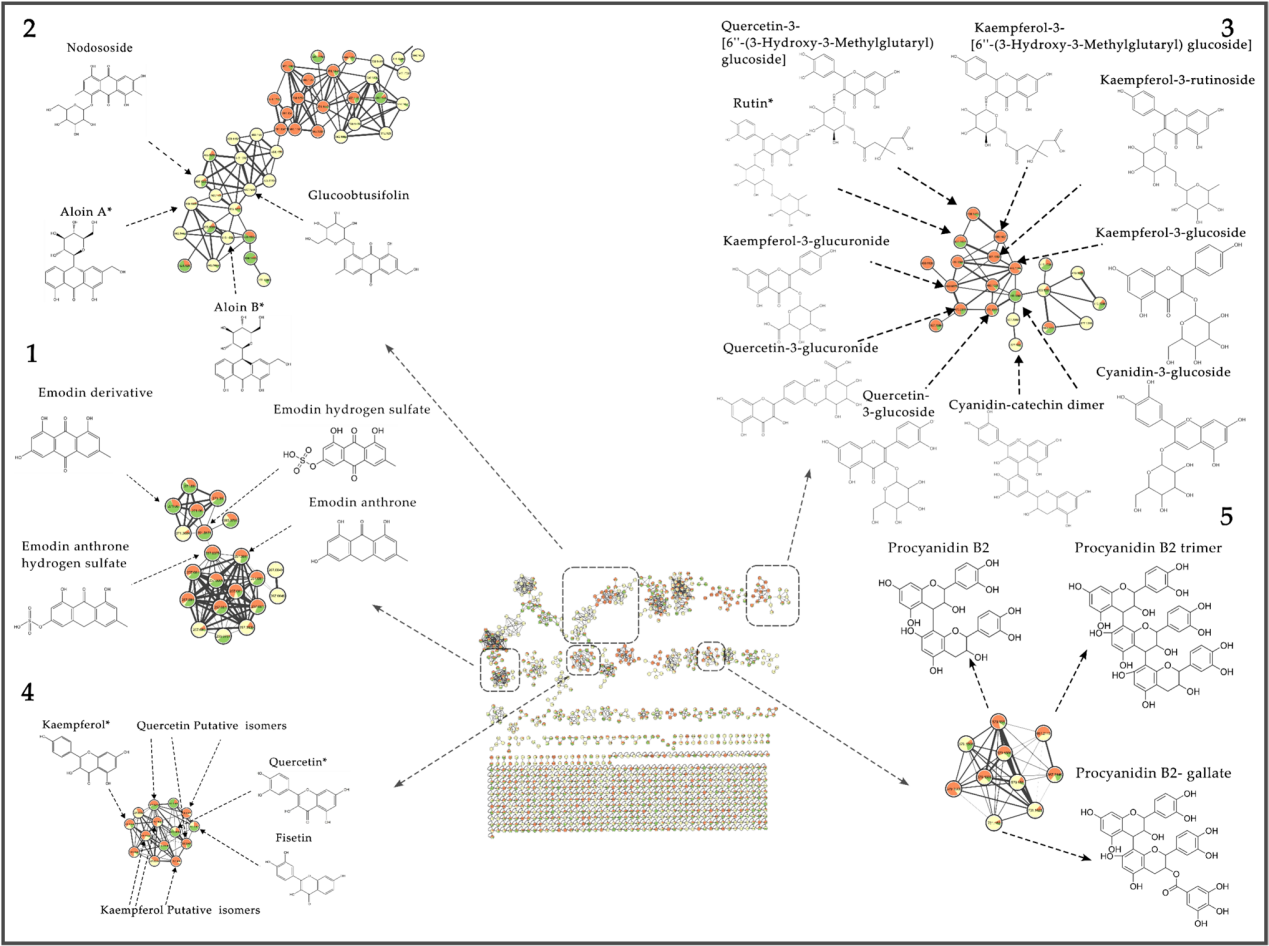
FBMN built with the ESI positive ionization data from *Rumex sanguineus* roots (yellow), leaves (orange) and stems (green). The edges serve as a graphical representation of the closeness or likeness in the mass spectrometry fragmentation patterns, providing a visual means to understand the relationships and similarities among the different compounds within the dataset. The annotated compounds clustered in the five sub-networks highlighted: (1) anthraquinones, (2) glycosylated anthraquinones, (3) polyphenols, (4) glycosylated polyphenols and (5) anthocyanidin. They are the main view a total of 1118 features (nodes), interconnected by 2060 edges, organized into 88 distinct clusters. *Level 1, metabolites confirmed with reference standards

Although the nature of our analysis does not permit conclusions about the absolute concentrations of compounds, it does reveal distribution trends. Notably, most of the annotated compounds (90%) are present in all three plant organs, as shown in the Venn diagram. (Fig. S5). However, what sets them apart is the variation in their proportions. In other words, a single compound found in all three organs can either be detected in all organs of *Rumex* but accumulate at different ratios (Fig. S6), or in some rare cases can be accumulated into a specific one (organ-specific). In our study, we observed that anthraquinones are generally accumulated in leaves, while aloin A, aloin B and some emodin derivatives, were predominantly found in the roots (Fig. 3). It is worth mentioning that many plant-specialized metabolites (anthraquinones and phenolic compounds) accumulate as glycosides as storage of bioactive molecules, hence, it is not surprising that aloin A and B were predominantly found in roots. These compounds are synthesized in the roots through the action of enzymes called glycosyltransferases (Yamada et al., 2020). However, further research is necessary to determine whether hydroxyanthracene glucoside derivatives are directly produced in the roots or if they are synthesized elsewhere in the plant and then stored in the roots. In the leaves, emodin appears to function as a deterrent to herbivores, especially vertebrates, at certain concentrations. Studies, (Trial & Dimond, 1979) suggest that emodin can be toxic to animals, which likely contributes to its role in protecting plants from herbivory. The glucosylation of emodin in the roots could be linked to the plant’s allelopathic strategy. By producing emodin glucosides, the plant may inhibit the growth of neighbouring plant species (Izhaki, 2002a). This allelopathic effect could give the plant a competitive advantage in crowded environments. While both biotic (e.g., interactions with other organisms) and abiotic (e.g., environmental factors) forces may influence the evolution of emodin, the distribution and concentration of emodin in various parts of a plant likely reflect a complex interplay of these factors. These interactions shape how emodin is produced, stored, and utilized across different species. Consequently, the presence of emodin in varying concentrations in different plant organs reflects not only the ecological conditions in which the plant thrives but also its diverse adaptive functions. Besides hydroxyanthracenes derivatives, it’s also important to consider the distribution of other bioactive compounds in plants, such as flavonols. While emodin glucosides are primarily concentrated in the roots, flavonols, including quercetin and kaempferol, tend to accumulate preferentially in the leaves. This is in line with the results reported for another *Rumex spp.*, namely *R. acetosella* (Feduraev et al., 2022)*.* Despite leveraging on advanced annotation tools, a significant proportion of features within *R. sanguineus* extracts, still remain unidentified, accounting for approximately 90% of the total.

### The molecular networking approach to decode the Rumex chemical composition

FBMN allowed the visualization of clusters of annotated compounds. The FBMN built with ESI positive data (Fig. 3) is a graphical representation of the relationships between chemical compounds, based on their spectral similarities. Each compound is represented by a node and the edges between the nodes indicate the similarity of their mass spectra on the basis of a modified cosine score. A total of 1118 features (nodes), interconnected by 2060 edges, were organized into 88 distinct clusters, while 507 remained without any connection indicating a lack of chemical class connection between them based on their shared fragmentation pathway with the used topology filter parameter settings to construct the molecular network. Table S1 provides a summary of all the annotated compounds clustered in the five sub-networks as thoroughly explained in the corresponding subsections (referred to as "sub-networks") highlighted in our analysis. The batch annotation spreadsheet can be found in the supplementary information as Table S1.

This spreadsheet has been made available on both Zenodo and MASSIVE platforms. It serves as a GNPS metadata-derived table, providing insights into molecule identities annotated through SIRIUS and manual compound annotation. By adopting a common machine-readable format, this resource offers valuable spectral annotation data to the scientific community. Additionally, all analysed fragmentation spectra can be accessed on MASSIVE in the form of mzXML files. Each annotated compound is associated with a distinct scan number, facilitating its retrieval within the molecular network uploaded on Zenodo.

### Sub-network exploration

Among all clusters depicted in Fig. 3, we focused on the two largest classes, namely anthraquinones and polyphenols, because of their significance in plant metabolism and their potential implications in various biological activities.

#### The anthraquinones sub-network: focus on emodin and its derivatives (1)

The anthraquinones family is largely present in roots, stems and leaves of *Rumex*; several anthraquinones derivatives were annotated thanks to their common fragmentation pattern. Starting with sub-network 1 (Figure S3 for ESI positive and Figure S4 for ESI negative), an emodin derivative was putatively annotated with feature number 6662 (*m/z* 271.0601, [M + H]^+^ ion adduct), based on the match of its fragmentation spectra with that of the emodin reference standard (Table S1, Supplementary Information). The substantial difference in their retention times (11.5 min for feature number 6662 vs 13.8 min of the reference standard, Table S1), indicates a different interaction of the compound with the stationary phase and a consequently difference in the distribution of emodin functional groups around the anthracene skeleton. At retention time of 12.7 min, we annotated emodin anthrone (feature number 7293) with [M + H]^+^ ion adduct of *m/z* 257.0811, and the data presented in Table S1, provides evidence of its characteristic fragmentation pattern as reported in library databases and literature (Bo et al., 2021). Notably, in this sub-network we annotated emodin anthrone hydrogen sulfate (feature number 5623, *m/z* 337.0375 [M + H]^+^ ion adduct, Table S1), based on the match between the fragmentation pattern of emodin anthrone, and to the characteristic hydrogen sulfite loss of 79.9578. Emodin hydrogen sulfate [M + H]^+^ ion adduct (*m/z* 351.0172), was putatively annotated with feature number 5708, at retention time of 9.8 min in the same sub-network (Table S1).

**Fig. 4:**
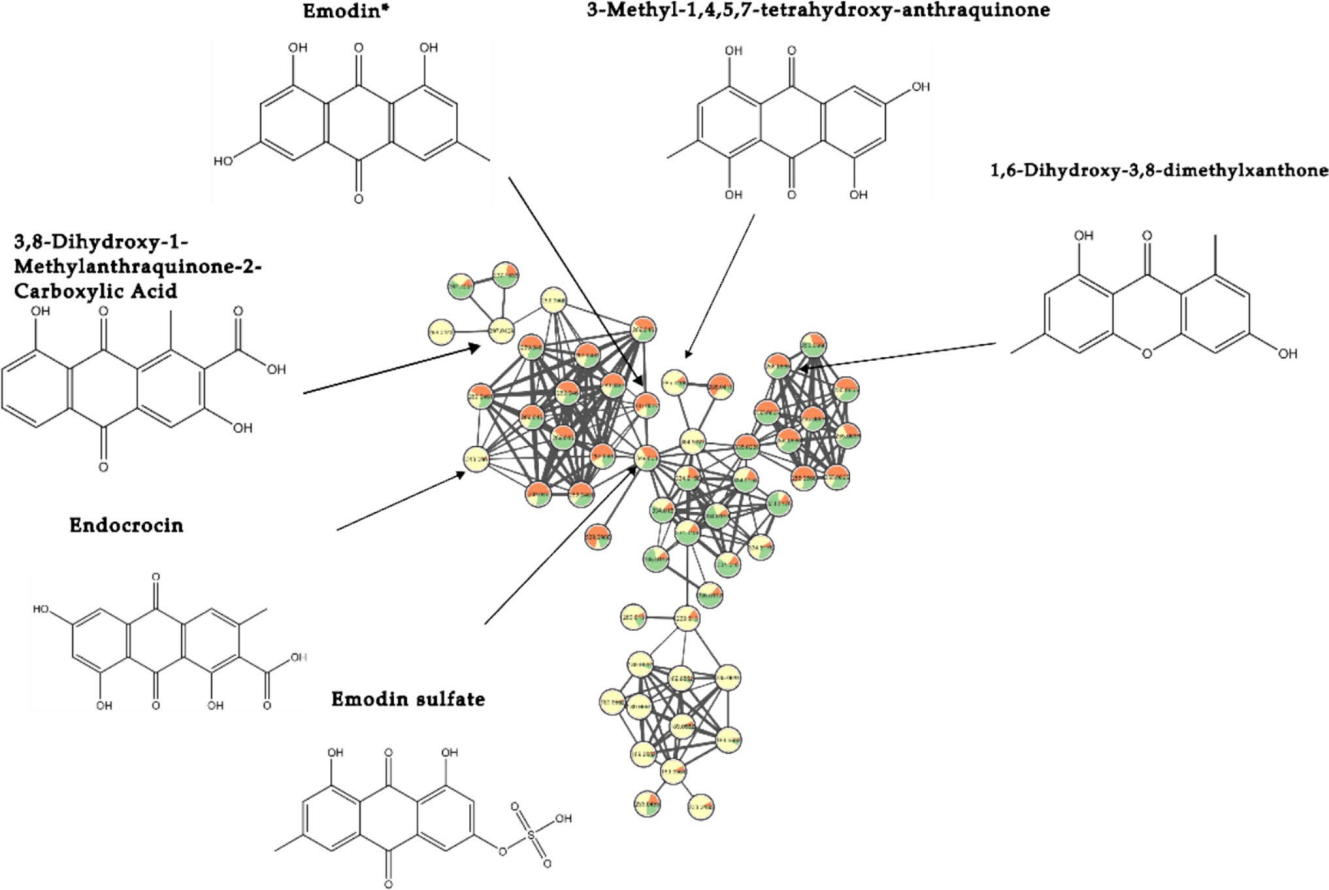
Anthraquinones sub-network obtained from ESI negative data. The chemical structure of emodin and other annotated derivatives are indicated. Color coding: roots (yellow), leaves (orange) and stems (green). *Level 1, metabolite confirmed with reference standards

**Fig. 5:**
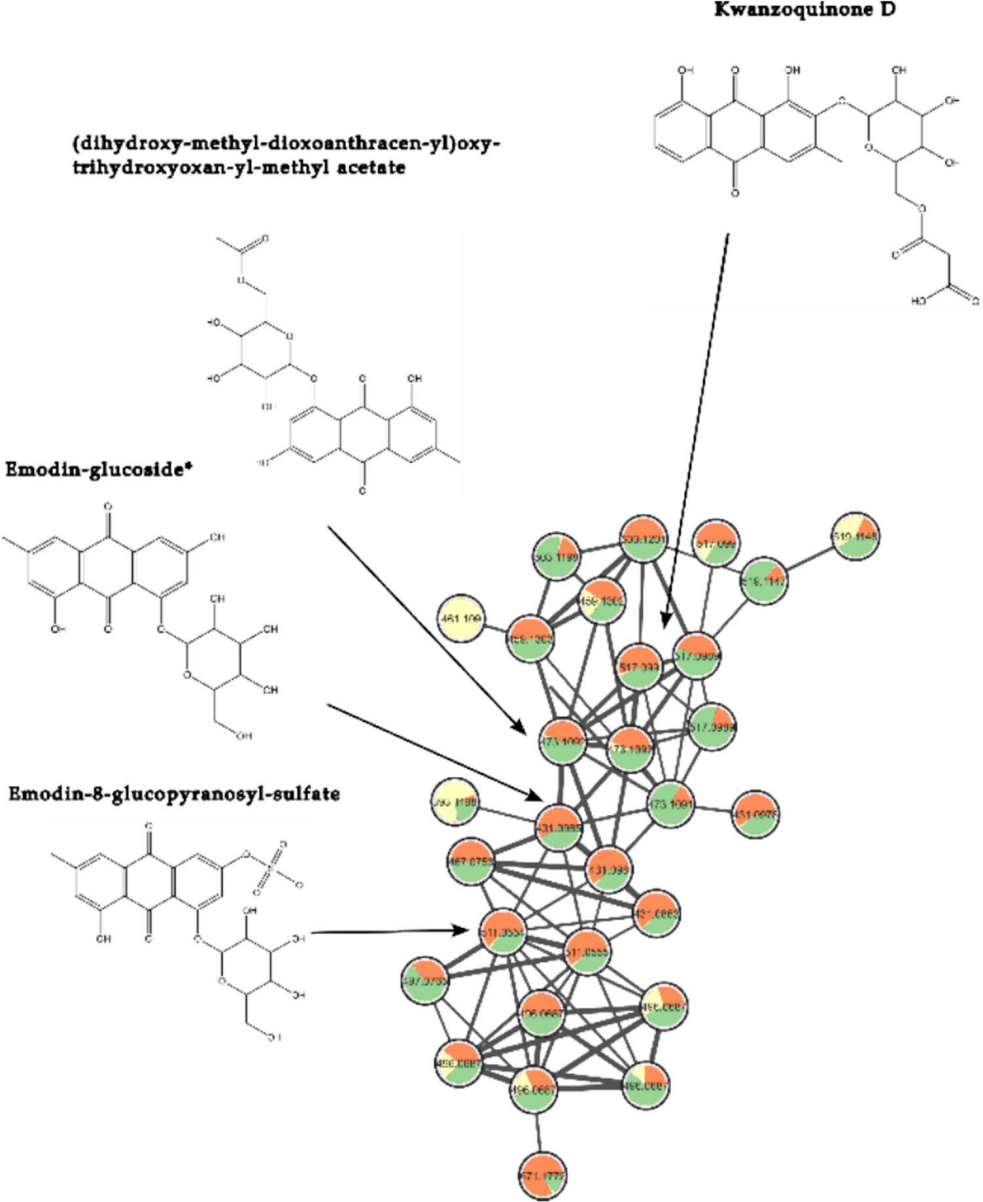
Sub-network of glycosylated anthraquinones (ESI negative data). The chemical structure of emodin-8-glucoside and other glycosides derivatives are indicated. Color coding: roots (yellow), leaves (orange) and stems (green). *Level 1, metabolite confirmed with reference standards

Due to their functional groups, anthraquinones easily produce [M–H]^−^ ion adducts in the negative ESI source. Therefore, the annotation of anthraquinones derivatives was conducted by investigating the ESI negative mode data (Zhao et al., 2018) (Fig. 4, 5). Among these, emodin stands out as a toxic compound (Younes et al., 2018). Given the coexistence of other emodin isomers with different retention times in our samples, particularly in the edible leaves, we aimed to confirm its presence with a reference standard. Emodin was unambiguously identified at retention time of 13.8 min with feature number of 10,182 (Table S1) and [M–H]^−^ ion adduct of *m/z* 269.0455. Moreover, compound with [M–H]^−^ ion adduct and *m/z* 285.0408 (Table S1), was annotated with feature number 10585 as a 3-methyl-1,4,5,7-tetrahydroxy-anthraquinone with a retention time of 14.96 min, indicating that it is an emodin derivative bearing a different rearrangement of the hydroxyl and methyl groups on the hydroxyanthracene scaffold. Dihydroxy-dimethylxanthone (Table S1) was tentatively identified with feature number 8935 at retention time of 12.9 min, and [M–H]^−^ ion adduct of *m/z* 255.0666; notably, its fragmentation spectra align with the characteristic fragmentation pattern observed for anthraquinones. Moreover, two carboxylic acid derivatives, namely dihydroxy- methylanthraquinone-carboxylic acid (feature number of 8702, rt 12.8 min, *m/z* 297.0409) and endocrocin (feature number of 7036, rt 11.5 min and *m/z* 313.0360) were annotated (Table S1). Endocrocin is an emodin derivative that forms a conjugate with a carboxylic acid, as evidenced by the fragmentation spectra uploaded on massIVE. The fragmentation pattern matches with that of emodin after the cleavage of the carboxylic acid, confirming the structural relationship between the two compounds. It is worth emphasizing that both compounds are predominantly concentrated in the roots (see yellow nodes in Fig. 4), indicating their specific accumulation in this plant organ.

Here, we introduce the negative molecular network of glycosylated anthraquinones (Fig. 5), which is closely linked to the previous network. Similar to free anthraquinones, anthraquinone conjugates easily produced [M–H]^−^ ion adduct in the negative ESI source and gave rise to [aglycone-H]^−^ ion as the MS/MS base peak by elimination of the glucosyl residue. At a retention time of 9.8 min, emodin-8-glucopyranosyl sulfate (Table S1) was detected with [M–H]^−^ ion adduct of *m/z* 511.0554 and feature number 5264. This compound can be considered the glycosylated corresponding ion of emodin hydrogen sulfate (Table S1). This may be attributed to the cleavage of the glucoside unit within the compound during the ESI ionization process (Es-Safi et al., 2007). Another noteworthy compound, emodin-8-glucoside, was identified (level I) with reference standard confirming its retention time at 10.7 min, feature number of 6115 and [M–H]^−^ ion adduct of *m/z* 431.0985. The structure elucidation in supplementary Table 1 highlights the loss of the glucoside unit and the subsequent fragmentation pattern typical of anthraquinones, aligning well with the reference standard. Another putative candidate was tentatively identified as dihydroxy-methyl-dioxoanthracen-yl)-oxy-trihydroxyoxan-yl-methyl acetate which is an emodin derivative. It is characterized by feature number 6998, a retention time of 11.6 min, and a [M–H]^−^ ion adduct with *m/z* 473.1092, as depicted in the fragmentation spectra uploaded on massIVE. Furthermore, the glucosyl derivative, kwanzoquinone D, (Table S1), was tentatively annotated with feature number 7183 at a retention time of 11.6 min and a [M–H]^−^ ion adduct of *m/z* 517.0990. It shares an identical fragmentation pattern with the previous candidate, but the key distinction lies in the presence of a carboxylic acid, which is bonded to the methyl acetate group in kwanzoquinone D (Cichewicz et al., 2002).

#### The anthraquinones sub-network: focus on aloin and its derivatives (2)

Sub-network number 2 (Fig. 3) was a focal point of our investigation due to its distinct composition of anthraquinone derivatives, notably the presence of aloin derivatives. Aloin A and aloin B were confirmed (level I) with reference standards with feature number of 5401 and 5188 respectively (Table S1), with a retention time of 9.2 min for aloin A and of 8.8 min for aloin B. Both of these compounds share the same [M + H]^+^ ion adduct of *m/z* 419.1336 and an identical fragmentation spectra since they are isomers. The distinguishing factor between the two spectra lies in the relative abundance of fragment ions *m/z* 239.0967 and *m/z* 211.0749. In aloin B, the former fragment is more prevalent than the latter, whereas in aloin A, these two fragments exhibit equivalent relative abundances. A compound directly associated with aloin B has an *m/z* of 463.1238 ([M + H]^+^ ion adduct). This compound has been tentatively annotated as nodososide, identified by feature number 4431 and a retention time of 7.6 min, as outlined in Table S1. The most abundant fragment is *m/z* 283.0600 which corresponds to the cleavage of the glucopyranoside unit (neutral loss of 180.0641) as indicated by the fragmentation spectra. The [M + H]^+^ ion adduct with a *m/z* of 447.1293, was putatively annotated as glucoobtusifolin (Table S1), feature number 5247 and retention time of 8.9 min. The fragmentation of glucoobtusifolin involves the same cleavage of a glucopyranoside unit, resulting in a neutral loss of 180.0641, as elucidated by the fragmentation spectra uploaded on MassIVE. This process leads to the generation of its most predominant fragment with an *m/z* ratio of 267.0650.

Overall, 37 anthraquinones were annotated (level II) in ESI positive (N = 19) and ESI negative (N = 18) modes, respectively, while 4 were identified (level I), namely emodin, emodin-8-glucoside, aloin A and aloin B. Emodin and emodin-8-glucoside exhibited highest accumulation in leaves, while other derivatives, such as carboxylic acid and sulfate derivatives, were predominantly accumulated in roots.

#### The polyphenols sub-network: focus on kaempferol and quercetin (3)

Phenolic compounds are ubiquitously distributed among plants, including various *Rumex* species, displaying substantial diversity in both type and concentration. Their presence serves multifaceted ecological and physiological functions across the plant genus (Feduraev et al., 2022). Firstly, kaempferol was annotated based on a [M + H]^+^ ion adduct (*m/z* 287.0553), with feature number 6917, and then confirmed with a reference standard by matching fragmentation spectra and retention times at 12.1 min (Table S1). The compounds closely connected with identified kaempferol (*m/z* 287.0553), have been annotated as kaempferol derivatives (class II) with feature numbers 4680, 4526 and 7816. The structure elucidation is based to the comparison of the feature fragmentation spectra to that of the reference standard. Their presence can be explained as in-source fragmentation of glycosylated forms of kaempferol, particularly feature 4680, whose aglycone retention time aligns with that of kaempferol-3-glucuronide. Additionally, feature 4526 could potentially be an in-source fragment derivative of kaempferol-3-glucoside, as evidenced by its matching retention time (7.6 min in kaempferol-3glucoside and 7.6 min in feature 4526) and the alignment of seven key fragments provides strong evidence of clear structural similarity (Barnes & Schug, 2011). Fisetin was putatively annotated (Table S1) within this sub network, with a feature number of 3601 (*m/z* 287.0552, [M + H]^+^ ion adduct) and, based on the similarity of its structure to that of kaempferol. The fragmentation spectra is similar to that of kaempferol, but the ions ratio is different (Chambers et al., 2012). Quercetin was also annotated (feature number 7757, *m/z* 303.0498*,* [M + H]^+^) and confirmed with reference standard (Table S1). Compounds with feature number and 4263 (rt 7.16 min) and feature 4057 (rt 6.88 min), were putatively annotated as quercetin isomers based on the similarity of their fragmentation spectra and the difference in their retention times as showed in on massIVE upload. Both features may be interpreted as an in-source fragments of a glycosylated forms of quercetin, as all annotated forms of quercetin exhibit the same retention times and given that elute earlier than the reference standard of quercetin (Barnes & Schug, 2011; Li et al., 2021).

#### The polyphenols sub-network: focus on kaempferol and quercetin glycosides (4)

Three quercetin derivatives, namely quercetin 3-[6″-(3-hydroxy-3-methylglutaryl) glucoside feature number 4505, *m/z* 609.1449), quercetin-3-glucuronide (feature number 4184, *m/z* 479.0819) and quercetin-3-glucoside (feature 4143, *m/z* 465.1028) were annotated in positive ESI mode ([M + H]^+^ ion adduct) as their MS/MS were predominated by the elimination of the sugar unit giving rise to the [aglicone + H]^+^ ion. In particular, the neutral losses of 306.1096 (162.0521 glucoside + 144.0437 methylglutaryl portion), 176.3270 (glucuronide) and 162.0521 (glucoside), respectively, enabled the annotation of the quercetin derivatives (Delcambre et al., 2015; Nagy et al., 2009). Feature number 4194 with an *m/z* of 957.1586 [M + H]^+^ ion adduct, was reported as a quercetin 3-glucuronide dimer (yet not annotated) formed in ESI source (Pan, 2008). Evidences are that quercetin 3-glucuronide and its dimer have the same retention time at 7.1 min; if the dimer is formed in-solution, the *m/z* of the monomeric species would be eluted at a different retention time with respect to the one of the dimeric species, due to the quite different molecular properties of monomer and dimer and the different interactions of the two species with the stationary phase of the column (Alcalde-Eon et al., 2007). Similarly, as for quercetin, four kaempferol derivatives were annotated based on the neutral losses. Feature number 5002 has close correlation to quercetin3-[6″-(3-hydroxy-3-methylglutaryl) glucoside (Table S1), thus was annotated as kaempferol 3-[6″-(3-hydroxy-3-methylglutaryl) glucoside (rt 8.54 min, *m/z* 593.1497, [M + H]^+^ ion adduct). Kaempferol is characterized by a precursor ion of *m/z* 287.0548 and as for the quercetin derivatives, the MS/MS shows the same neutral loss of 306.0952 (feature 4505) consistent with the cleavage of the sugar unit bonded to 3-hydroxy-3-methylglutaryl sub-unit. Along with its congeners, the ion *m/z* of 449.1087 (feature 4533) was annotated as kaempferol-3-glucoside; within the fragmentation spectra, the glucoside unit produces a neutral loss of 162.0521 while the aglycone unit confirms kaempferol structure (precursor ion of *m/z* 287.0548). Furthermore, kaempferol-3-rutinoside (feature number 4513, *m/z* 595.1653, [M + H]^+^ ion adduct) was annotated as a disaccharide form of kaempferol. As for the other glycosylated forms, we can observe a neutral loss (in this case of 308.1114) corresponding to the O-glycoside portion bonded to kaempferol. Feature number 4660 was tentatively annotated as kaempferol-3-glucuronide with a rt of 7.98 min and *m/z* of 463.0874 [M + H]^+^. Feature number 4039 (*m/z* 611.1607, [M + H]^+^ ion adduct) was unambiguously identified as rutin with the reference standard and confirmed with the rt (6.7 min, Table S1). Cyanidin-3-glucoside (feature 2095, *m/z* 449.1085, [M]^+^) was tentatively identified monitoring the flavylium glycosylated cation (Barnes & Schug, 2011) and the unique fragment ion of *m/z* 149.0232, which enable to distinguish cyanidin derivatives from quercetin and kaempferol. Based on a similar assumption, feature 4302 (*m/z* 577.1337, [M]^+^ ion adduct, rt 7.2 min) was putatively described as a cyanidin-catechin dimer This identification is supported by the observed neutral loss of 290.0799 corresponding to the loss of catechin, and by the fact that cyanidin mass spectra are dominated by the molecular ion [M] + rather than [M + H] + due to anthocyanins existing as flavylium cations under acidic conditions (Li et al., 2021). This conclusion is further corroborated by the fragmentation spectra (uploaded to MassIVE) as described in the literature (Delcambre et al., 2015; Nagy et al., 2009).

#### The anthocyanidin sub-network: focus on proanthocyanidins (5)

Previous literature reported high content of proanthocyanidins (Spencer et al., 2007) in *R. obtusifolius* (Sganzerla et al., 2019), hence we aimed to verify their presence also in *R. sanguineus*. An unequivocal network emerged, including proanthocyanidin B2, proanthocyanidin B2-gallate, and a trimer of proanthocyanidin B2 (Fig. 3). Proanthocyanidin B2, was putatively annotated (feature number 1695, rt 3.9 min, *m/z* 579.1510, [M + H]^+^) based on the fragmentation pattern, which indicates a potential cleavage corresponding to an epicatechin (a single monomer forming proanthocyanidin B2). This results in the formation of the fragment ion *m/z* 287.0480. Similarly, proanthocyanidin B2-gallate was annotated (feature number of 3328, rt 5.5 min, *m/z* 731.1621, [M + H]^+^) matching his fragments with two neutral losses for the cleavage of propyl gallate (neutral loss of 152.0528 and of 292.0953 leading to the formation of the *m/z* 287.0480 as reported in literature (Rue et al., 2018)). Furthermore, we proposed the annotation of a proanthocyanidin B2 trimer, characterized by the presence of three individual epicatechin monomers connected via C–C bonds. This trimeric compound (feature number 2108, rt 4.3 min, *m/z* 867.2146, [M + H]^+^) is subject to similar fragmentation pattern, with two epicatechin cleavages and consequently neutral losses of 292.0953 as reported in massIVE upload. As indicated in the literature, the occurrence of trimers is more widespread than commonly thought. A-type trimers (Li et al., 2021) are commonly found in cranberry (Foo et al., 2000), while trimeric and tetrameric proanthocyanidins have been reported in the aerial parts of *Rumex acetosa* (Derksen et al., 2014).

### Quantitative analysis of emodin and emodin-8-glucoside in roots, stems and leaves

**Fig. 6:**
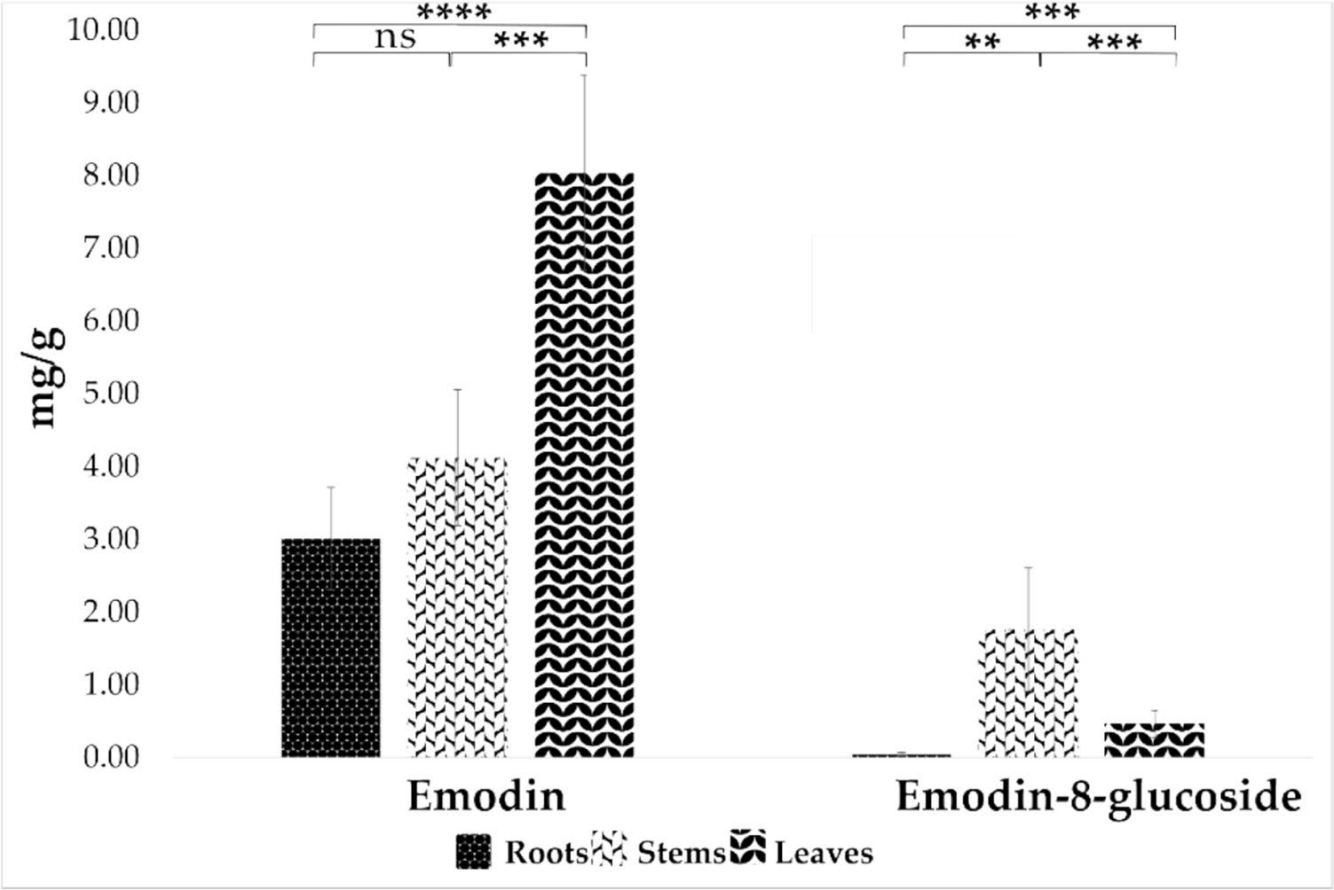
Emodin and emodin-8-glucoside content across the three different organs. The p-value was calculated to assess the statistical significance of differences between roots, stems and leaves

Our metabolomic analysis showed that several anthraquinones (19 annotated in ESI positive ionization mode and 18 annotated in ESI negative), including emodin and emodin-8-glucoside, are differently distributed across the three different plant organs of *Rumex*. Despite the lack of precise concentration data, we observed that these compounds are present in varying amounts in each organ, highlighting specific patterns of accumulation. Because of their know toxicity (Paneitz et al., 1999) and the recent EFSA opinion on the presence of emodin and aloe-emodin in edible plants (Younes et al., 2018) a quantitative analysis was performed. As shown in Fig. 6A the content of emodin was significantly (p < 0.0001) higher in leaves (8 mg/g of dry weight), compared to stems and roots (leaves > stems > roots) considering an adult growth stage and a winter harvesting period. A different accumulation trend was observed for emodin-8-glucoside (Fig. 6A), whose content was significantly higher (p < 0.0005) in stems (2 mg/g of dry weight) followed by leaves and roots (stems > leaves > roots). This difference across organs distribution may be elucidated considering that emodin-8-glucoside serves as a storage form of emodin. Activation of this storage form involves the action of glycosyltransferases, which facilitate the removal of a sugar unit from the anthraquinone ring. The observed variation in distribution across plant organs, with potentially higher expression in stems, suggests the involvement of glycosyltransferase activity. However, further studies are necessary to conclusively affirm this hypothesis (Izhaki, 2002b; Paneitz et al., 1999).

Considering that leaves constitute the edible part of the plant and that young leaves are the most suitable for eating consumption, we quantified the emodin content in leaves at distinct growth stages (young and adult leaves). When comparing the levels of emodin between young and adult leaves, a notable difference was observed: the concentration of emodin was found to be significantly higher in young leaves (Fig. S7) showing an increase of approximately 30% compared to adult leaves.

This result could be explained considering that synthesis and accumulation of secondary metabolites in plants is affected the growing stage and by abiotic environmental factors, such as light intensity, soil minerals, osmotic stresses (drought and salinity) and seasonality (Izhaki, 2002b). Studies supporting this hypothesis indicate that emodin levels in plants depend on season and light intensity. The leaves of *Rheum undulatum* in Europe exhibited the highest concentration of total anthraquinones, with approximately 50% being emodin, during the spring season (April). Subsequently, there was a consistent decline in anthraquinone levels throughout the summer, reaching their lowest amounts in late summer (September). This observed seasonal variation could be indicative of a trade-off between the plant’s developmental processes and its defence mechanisms (Paneitz et al., 1999).

The accumulation of emodin, emodin-8-glucoside and other derivatives such as aloin A and B, has been reported also for other *Rumex* species, such as *R. confertus, R. crispus, R. obtusifolius* and *R. aquaticus* (Eom et al., 2020; Pelzer et al., 2022; Poluyanov et al., 2022). Our findings correspond well within the wide range documented in existing literature (2 to 160 mg/g) (Wegiera et al., 2007), despite the considerable variability in emodin content observed across seasons, organs, and *Rumex* species. The variability of emodin concentration in the roots shows significant differences among various Rumex species. In certain species, such *as R. acetosella, R. confertus, and R. crispus*, the emodin content is higher in the roots than in the leaves. Conversely, in other species, the emodin levels are higher in the leaves. Notably, in *R. obtusifolius*, the proportional distribution aligns with our findings, with emodin levels being higher in the leaves compared to the roots.

Given the absence of *R. sanguineus* from EFSA’s plant list containing approved hydroxyanthracene derivatives in food supplements, our research gains heightened significance. The EFSA opinion classifies hydroxyanthracene derivatives (including emodin, aloin and derivatives) as potentially genotoxic and carcinogenic. Notably, EFSA faces challenges in providing precise recommendations for the daily intake of hydroxyanthracene derivatives that would not raise concerns regarding potential adverse health effects. This underscores the need for continued investigation and assessment of the potential implications associated with the presence of hydroxyanthracene derivatives in *R. sanguineus*. As *R. sanguineus* demonstrates the ability to biosynthesize anthraquinone, particularly emodin and its derivatives, the intricate and at times contradictory pharmacological activities associated with emodin necessitate thorough investigation. In 2021 the European Commission considered the significant adverse health effects linked to the consumption of aloe-emodin, emodin, danthron, and aloe extracts containing hydroxyanthracene derivatives in food, and given the absence of a defined daily intake level that does not raise concerns for human health, prohibited these substances (Annex III, Part A of Regulation (EC) No 1925/20069) (L96, 2021).

The implementation of non-targeted metabolomics in our investigation has enhanced our understanding of the chemical profile of *R. sanguineus* highlighting its potential for applications in contemporary nutrition and agriculture. From a food nutritional and safety perspective, *Rumex* leaves were found to be rich in bioactive compounds, including phenolic, coumarins and cinnamic acids. When it comes to safety aspects, cooking *Rumex* leaves prior to consumption is expected to mitigate concerns regarding emodin content, as thermal processing induces the degradation of emodin (Narayanan et al., 2015). However, rigorous analytical investigations are essential to elucidate the kinetics of emodin degradation under various cooking conditions, thereby facilitating informed decision making to determine the safety and nutritional value of cooked *Rumex* leaves.

## Supplementary Information

Below is the link to the electronic supplementary material.

Supplementary file1 (DOCX 4208 KB)

## Data Availability

The positive FBMN is available at: https://gnps.ucsd.edu/ProteoSAFe/status.jsp?task=f3363189286c428bae3470d9fbd371c6.The negative FBMN is available at: https://gnps.ucsd.edu/ProteoSAFe/status.jsp?task=4e3d798eb99e42a58f0401ca78399186. LC-HRMS dataset that support the findings of this study have been deposited in MASSIVE: https://massive.ucsd.edu/ProteoSAFe/dataset.jsp?accession=MSV000092024. The MZmine batch file, alongside comprehensive Excel tables containing 449 annotated compounds in both ESI positive and ESI negative modes, as well as the detailed table of sub-networks analysed, have been uploaded to Zenodo for accessibility and reference: https://doi.org/10.5281/zenodo.14236385.
