# Supplementary material for: Non-targeted metabolomics-based molecular networking enables the chemical characterization of Rumex sanguineus, a wild edible plant
Source: Metabolomics. 2025 Jan 24;21(1):19. doi: 10.1007/s11306-024-02210-2 (PMC11761831; doi:10.1007/s11306-024-02210-2)
Supplement: Supplementary file 1 — Supplementary file1 (DOCX 4208 KB) [file 11306_2024_2210_MOESM1_ESM.docx]

**Supporting Information for:**

**Non-targeted metabolomics-based molecular networking enables the chemical characterization of Rumex sanguineus**

Valentina Ramundi^1,2,3^, Mitja M. Zdouc^4^, Justin J.J. van der Hooft^4,5^, Enrica Donati^2^, Sara Cimini^3^, Laura Righetti^1,6 *^

^1^ *Laboratory of Organic Chemistry, Wageningen University & Research, 6708 WE Wageningen, the Netherlands.*

*^2^ Institute for Biological System (ISB), National Research Council of Italy (CNR), via Salaria km 29.300, 00015 Monterotondo Scalo (Rome), Italy*

*^3^ Department of Science and Technology for Humans and the Environment, Campus Bio-Medico University of Rome, Via Álvaro del Portillo, 21, Rome,00128, Italy.*

*^4^ Bioinformatics Group, Wageningen University & Research, Droevendaalsesteeg 1, 6708 PB, Wageningen, the Netherlands****.***

*^5^ Department of Biochemistry, University of Johannesburg, 2006 Johannesburg, South Africa.*

*^6^ Wageningen Food Safety Research, Wageningen University & Research, 6700 AE, the Netherlands****.***


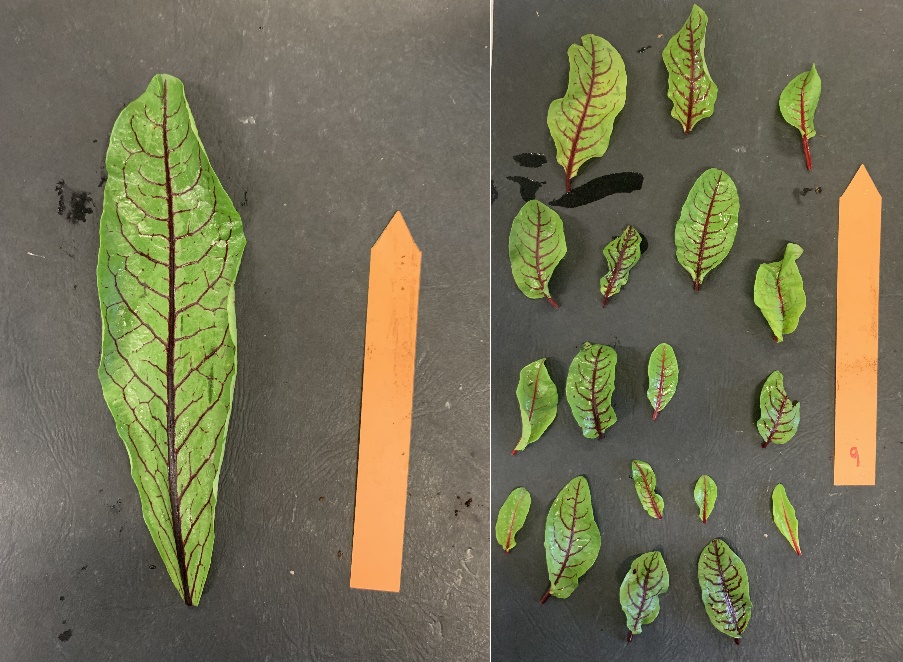


**Figure S1.** Adult and young leaves sampling. To ensure accurate measurements of leaf area and length during Fiji (ImageJ) analysis, we utilized an orange bookmark (1.8 cm by 14.3 cm) as a scale.


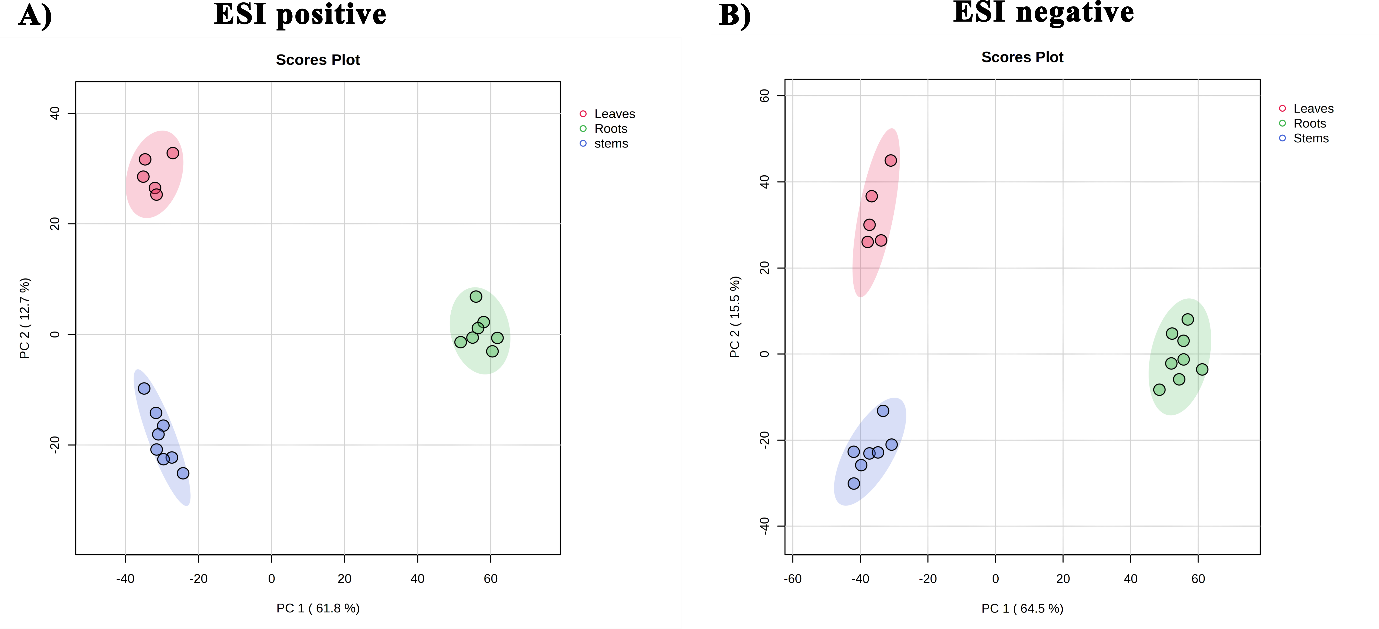


**Figure S*2.*** Principal component analysis (PCA) models of the first two principal components (PCs) built from (A) positive (R^2^ = 0.74) and (B) negative (R^2^ = 0.80) ionization data. Colour coding: roots (green), leaves (red) and stems (blue). In both positive and negative ionization mode, the first PC explains most of the variance (62% and 65%, respectively) and separates roots from stems and leaves, while the second PC discriminates the finer differences between leaves and stems.


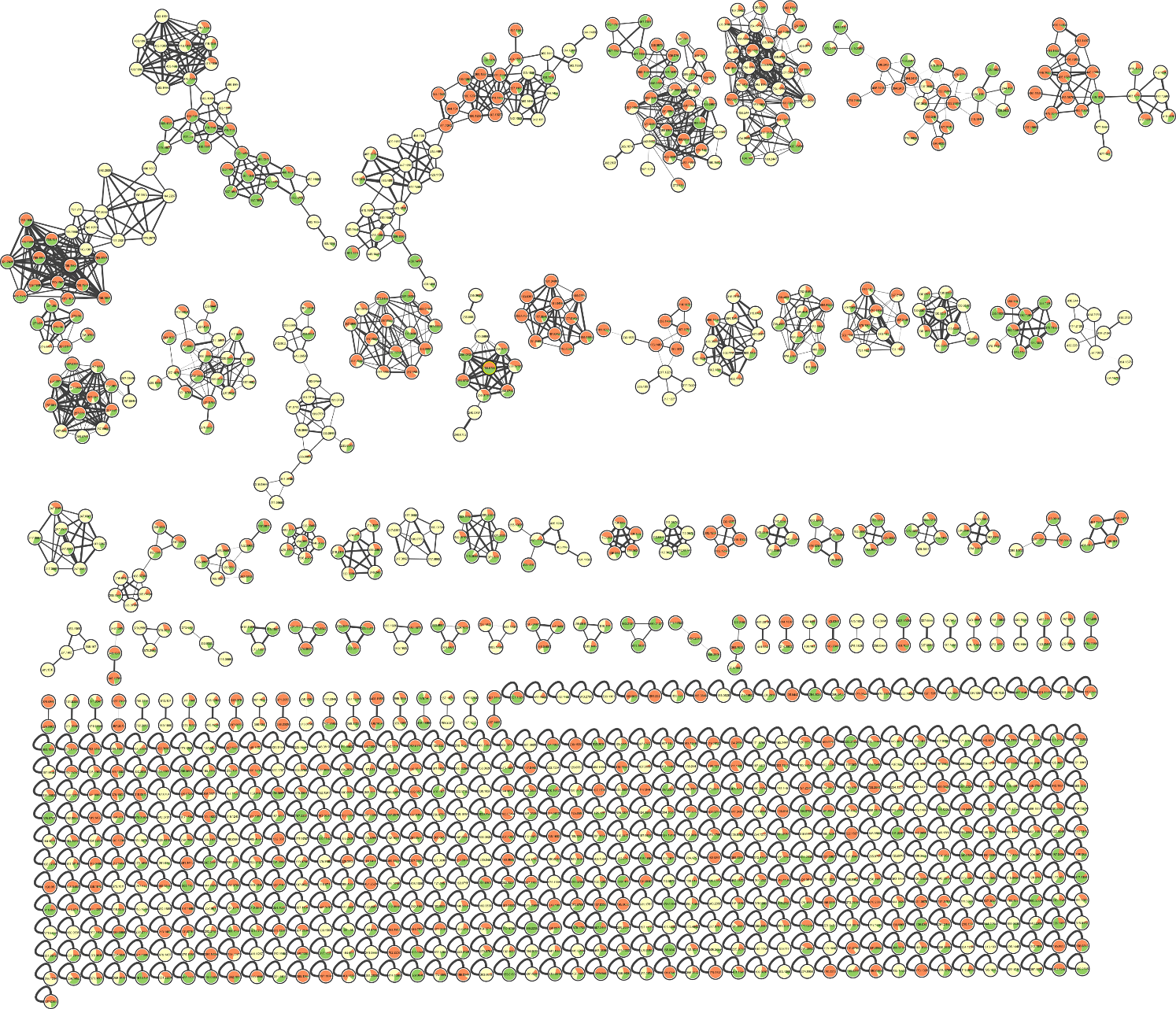


**Figure S3.** FBMN built with ESI positive ionization mode data form Rumex sanguineus roots (yellow), leaves (orange) and stems (green). A total of 1118 features (nodes), interconnected by 2060 edges, were organized into 88 distinct clusters.


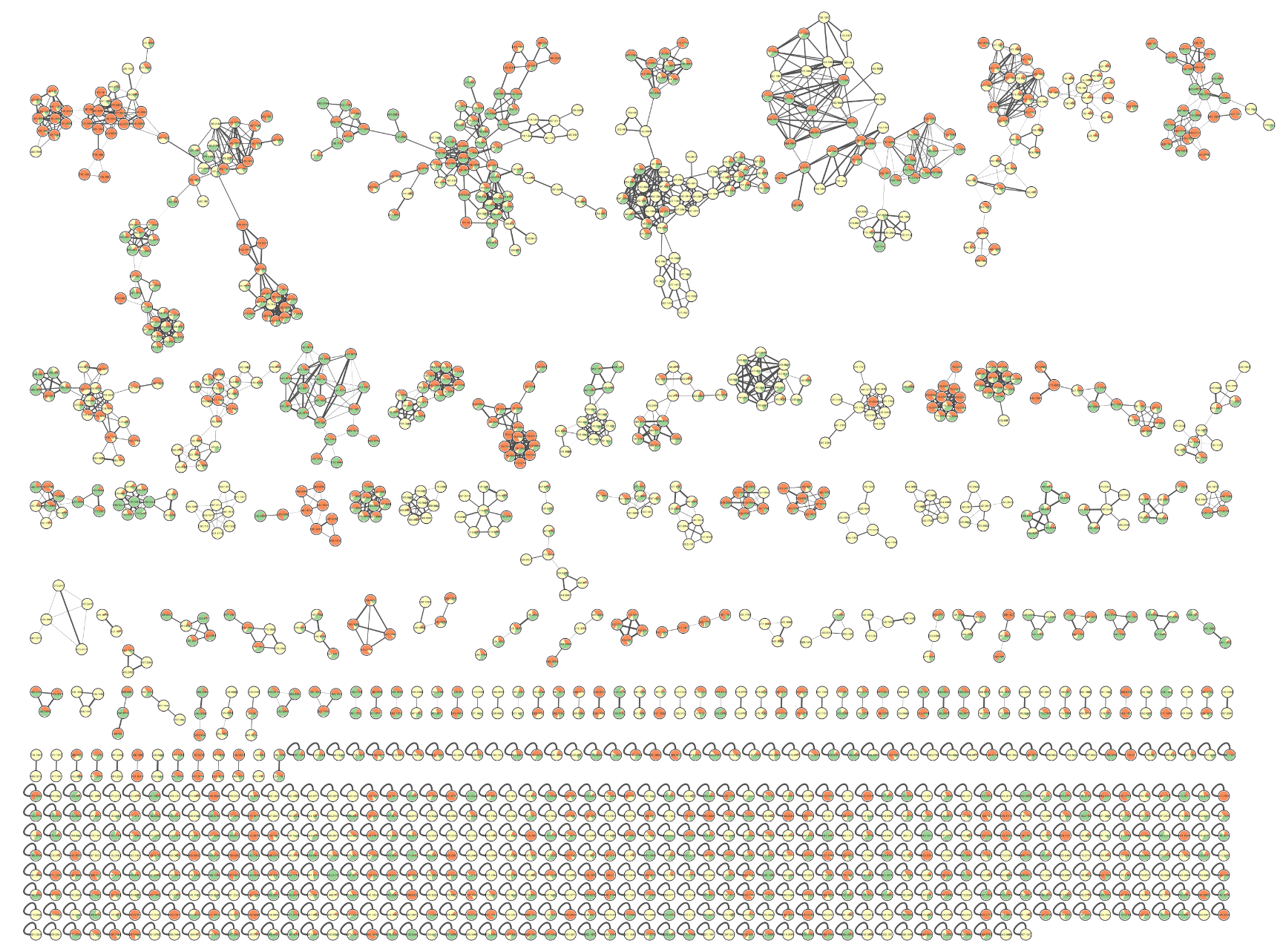


**Figure S4.** FBMN built with ESI negative ionization mode data form Rumex sanguineus roots (yellow), leaves (orange) and stems (green). A total of 1417 features (nodes), interconnected by 2527 edges, were organized into 128 distinct clusters.


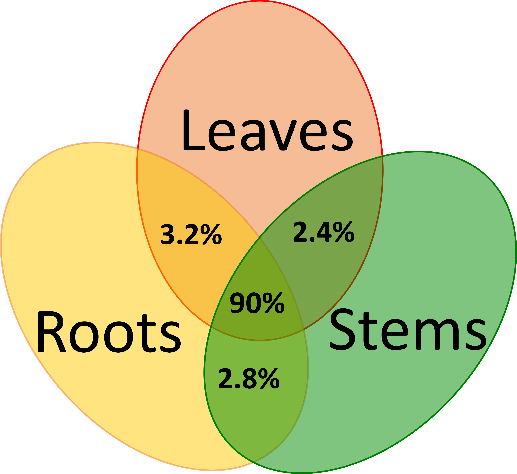


**Figure S5.** Venn diagram displaying the distribution of 347 annotated compounds in roots, stems and leaves. As it can been seen from the figure, most compounds (90%) are common to the three plant organs, even though their accumulation can significantly change between roots, stems and leaves.


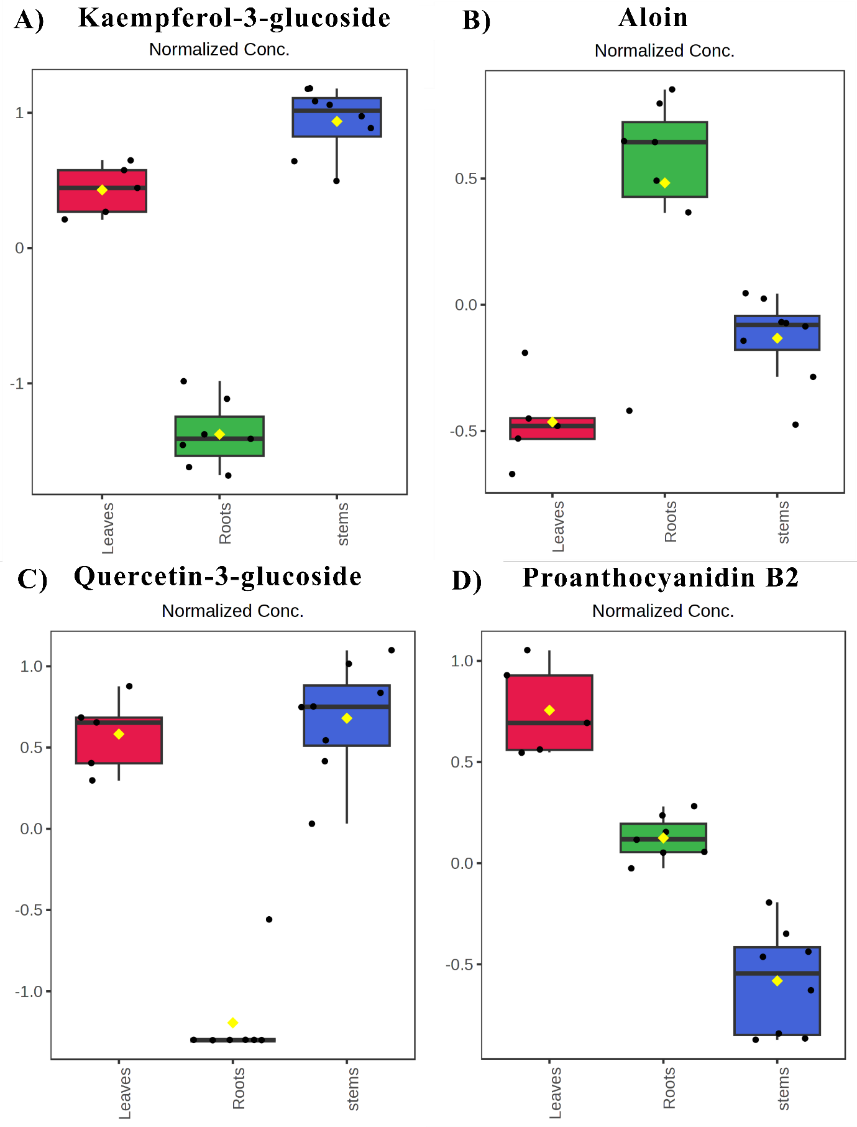


**Figure S6.** Accumulation of (A) kaempferol-3-glucoside, (B) aloin, (C) quercetin-3-glucoside and (D) proanthocyanidin B2 in leaves (red), roots (green) and stems (blue) groups.


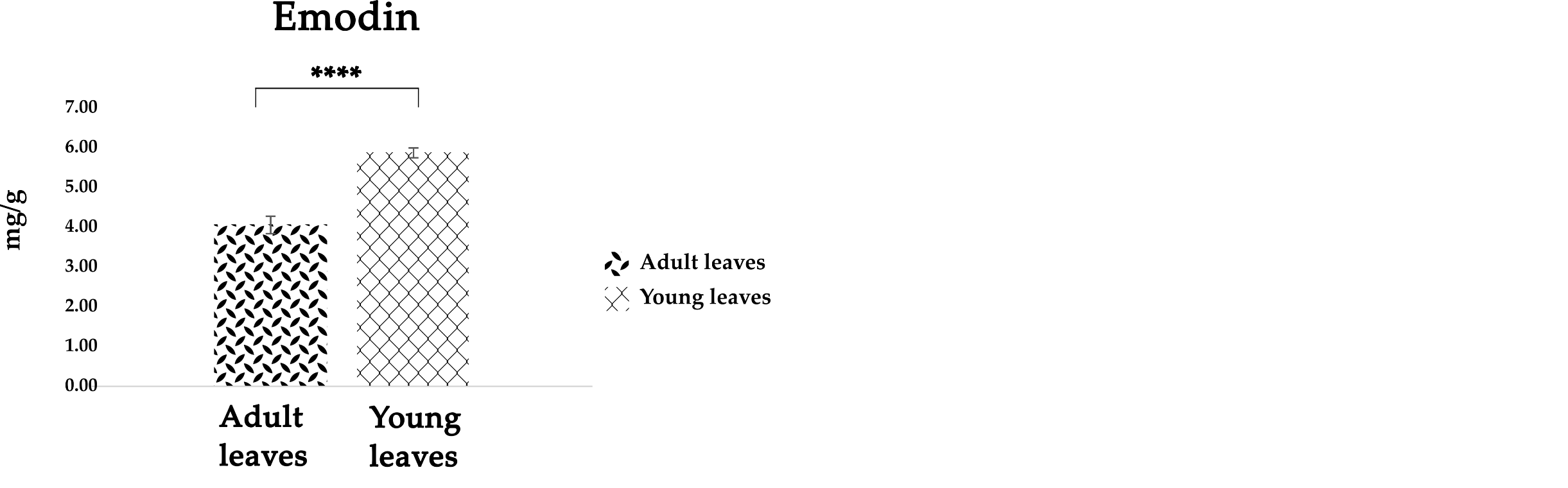


**Figure S7.** Emodin content in leaves across two different growth stages (sampling August 2022); leaves were classified as adult and young based on their length and areas as indicated in Figure S1.

**Fragmentation spectra**

**Table 1****.** This table includes all annotated compounds clustered into the five sub-networks, as thoroughly explained in the corresponding subsections of our analysis. Batch mode library creation supports and streamlines reference library formation, reducing errors, especially when handling a large number of spectra. All data are available on massIVE in mzXML format.

| FILENAME | COMPOUND  NAME | MOLECULE  MASS | INSTRUMENT | ION  SOURCE | EXTRACT  SCAN | CHARGE | ION  MODE | ACQUISITION | EXACT  MASS | DATA  COLLECTOR | ADDUCT | LIB  QUALITY | PI |
| --- | --- | --- | --- | --- | --- | --- | --- | --- | --- | --- | --- | --- | --- |
| 5L2_POS.mzXML | Emodin derivative | 271.0600 | Orbitrap | LC-ESI | 6662 | 1 | Positive | crude | 270.0523 | Valentina_Ramundi | M+H | 3 | Laura_Righetti |
| 6S2_POS.mzXML | Emodin_anthrone derivative | 257.0811 | Orbitrap | LC-ESI | 7293 | 1 | Positive | crude | 256.0730 | Valentina_Ramundi | M+H | 3 | Laura_Righetti |
| 8L2_POS.mzXML | Emodin-anthrone-hydrogen-sulfate | 337.0374 | Orbitrap | LC-ESI | 5623 | 1 | Positive | crude | 336.0298 | Valentina_Ramundi | M+H | 3 | Laura_Righetti |
| 8S2_POS.mzXML | Emodin-hydrogen-sulfate | 351.0172 | Orbitrap | LC-ESI | 5708 | 1 | Positive | crude | 350.0090 | Valentina_Ramundi | M+H | 3 | Laura_Righetti |
| 5R2_POS.mzXML | 3-Methyl-1,4,5,7-tetrahydroxy-anthraquinone | 285.0408 | Orbitrap | LC-ESI | 10585 | 1 | Negative | crude | 286.0483 | Valentina_Ramundi | M-H | 3 | Laura_Righetti |
| 8L1_POS.mzXML | Dihydroxy-dimethylxanthone | 255.0664 | Orbitrap | LC-ESI | 8935 | 1 | Negative | crude | 256.0741 | Valentina_Ramundi | M-H | 3 | Laura_Righetti |
| 7R1_POS.mzXML | 3,8-Dihydroxy-1-Methylanthraquinone-2-Carboxylic Acid | 297.0409 | Orbitrap | LC-ESI | 8702 | 1 | Negative | crude | 298.0483 | Valentina_Ramundi | M-H | 3 | Laura_Righetti |
| 7R1_POS.mzXML | Endocrocin | 313.0359 | Orbitrap | LC-ESI | 7036 | 1 | Negative | crude | 314.0432 | Valentina_Ramundi | M-H | 3 | Laura_Righetti |
| 8L2_POS.mzXML | Emodin-8-glucopyranosyl sulfate | 511.0552 | Orbitrap | LC-ESI | 5264 | 1 | Negative | crude | 512.0630 | Valentina_Ramundi | M-H | 3 | Laura_Righetti |
| 8L2_POS.mzXML | Emodin 1-O-beta-D-glucoside | 431.0984 | Orbitrap | LC-ESI | 6115 | 1 | Negative | crude | 432.1062 | Valentina_Ramundi | M-H | 3 | Laura_Righetti |
| 8L2_POS.mzXML | Dihydroxy-methyl-dioxoanthracen-yl)-oxy-trihydroxyoxan-yl-methyl acetate | 473.1087 | Orbitrap | LC-ESI | 6998 | 1 | Negative | crude | 474.1176 | Valentina_Ramundi | M-H | 3 | Laura_Righetti |
| 8L2_POS.mzXML | Kwanzoquinone_D | 517.0988 | Orbitrap | LC-ESI | 7183 | 1 | Negative | crude | 518.1065 | Valentina_Ramundi | M-H | 3 | Laura_Righetti |
| 8R2_POS.mzXML | Aloin_B | 419.1328 | Orbitrap | LC-ESI | 5188 | 1 | Positive | crude | 418.1269 | Valentina_Ramundi | M+H | 3 | Laura_Righetti |
| 8R2_POS.mzXML | Aloin-A | 419.1328 | Orbitrap | LC-ESI | 5401 | 1 | Positive | crude | 418.1269 | Valentina_Ramundi | M+H | 3 | Laura_Righetti |
| 5R2_POS.mzXML | Nodososide | 463.1238 | Orbitrap | LC-ESI | 4431 | 1 | Positive | crude | 462.1676 | Valentina_Ramundi | M+H | 3 | Laura_Righetti |
| 5R2_POS.mzXML | Glocoobtusifolin | 447.1290 | Orbitrap | LC-ESI | 5247 | 1 | Positive | crude | 446.1218 | Valentina_Ramundi | M+H | 3 | Laura_Righetti |
| 5L2_POS.mzXML | Kaempferol | 287.0550 | Orbitrap | LC-ESI | 6917 | 1 | Positive | crude | 286.0483 | Valentina_Ramundi | M+H | 3 | Laura_Righetti |
| 5S1_POS.mzXML | Fisetin | 287.0552 | Orbitrap | LC-ESI | 3601 | 1 | Positive | crude | 286.0483 | Valentina_Ramundi | M+H | 3 | Laura_Righetti |
| 8S1_POS.mzXML | Quercetin derivative | 303.0500 | Orbitrap | LC-ESI | 7757 | 1 | Positive | crude | 302.0432 | Valentina_Ramundi | M+H | 3 | Laura_Righetti |
| 5L2_POS.mzXML | Kaempferol_3-[6''-(3-hydroxy-3-methylglutaryl) glucoside | 593.1498 | Orbitrap | LC-ESI | 5002 | 1 | Positive | crude | 592.1438 | Valentina_Ramundi | M+H | 3 | Laura_Righetti |
| 5L2_POS.mzXML | Quercetin_3-glucuronide | 479.0821 | Orbitrap | LC-ESI | 4184 | 1 | Positive | crude | 478.0753 | Valentina_Ramundi | M+H | 3 | Laura_Righetti |
| 5L2_POS.mzXML | Quercetin_3-glucoside | 465.1036 | Orbitrap | LC-ESI | 4143 | 1 | Positive | crude | 464.0960 | Valentina_Ramundi | M+H | 3 | Laura_Righetti |
| 5L2_POS.mzXML | Quercetin_3-[6’’-(3-hydroxy-3-methylglutaryl) glucoside | 609.1449 | Orbitrap | LC-ESI | 4505 | 1 | Positive | crude | 608.1383 | Valentina_Ramundi | M+H | 3 | Laura_Righetti |
| 5L2_POS.mzXML | Kaempferol_3-glucoside | 449.1079 | Orbitrap | LC-ESI | 4533 | 1 | Positive | crude | 448.1011 | Valentina_Ramundi | M+H | 3 | Laura_Righetti |
| 5L2_POS.mzXML | Kaempferol_3-rutinoside | 595.1658 | Orbitrap | LC-ESI | 4513 | 1 | Positive | crude | 594.1590 | Valentina_Ramundi | M+H | 3 | Laura_Righetti |
| 5L2_POS.mzXML | Kaempferol_3-glucuronide | 463.0874 | Orbitrap | LC-ESI | 4660 | 1 | Positive | crude | 462.0804 | Valentina_Ramundi | M+H | 3 | Laura_Righetti |
| 5L2_POS.mzXML | Rutin | 611.1605 | Orbitrap | LC-ESI | 4039 | 1 | Positive | crude | 610.1539 | Valentina_Ramundi | M+H | 3 | Laura_Righetti |
| 5S1_POS.mzXML | Cyanidin-3-glucoside | 449.1083 | Orbitrap | LC-ESI | 2095 | 1 | Positive | crude | 449.1083 | Valentina_Ramundi | M+ | 3 | Laura_Righetti |
| 6R1_POS.mzXML | Cyanidin-catechin conjugate | 577.1337 | Orbitrap | LC-ESI | 4302 | 1 | Positive | crude | 576.1273 | Valentina_Ramundi | M+H | 3 | Laura_Righetti |
| 8L2_POS.mzXML | Proathocyanidin_B2 | 579.1500 | Orbitrap | LC-ESI | 1695 | 1 | Positive | crude | 578.1423 | Valentina_Ramundi | M+H | 3 | Laura_Righetti |
| 7L2_POS.mzXML | Proathocyanidin_B2-gallate | 731.1607 | Orbitrap | LC-ESI | 3328 | 1 | Positive | crude | 730.1539 | Valentina_Ramundi | M+H | 3 | Laura_Righetti |
